# Supplementary material for: New Insights into the Genetic Basis of Lysine Accumulation in Rice Revealed by Multi-Model GWAS
Source: Int J Mol Sci. 2024 Apr 25;25(9):4667. doi: 10.3390/ijms25094667 (PMC11083390; doi:10.3390/ijms25094667)
Supplement: Supplementary file 1 [file ijms-25-04667-s001.zip › Supplementary Figure S1-7.pdf]

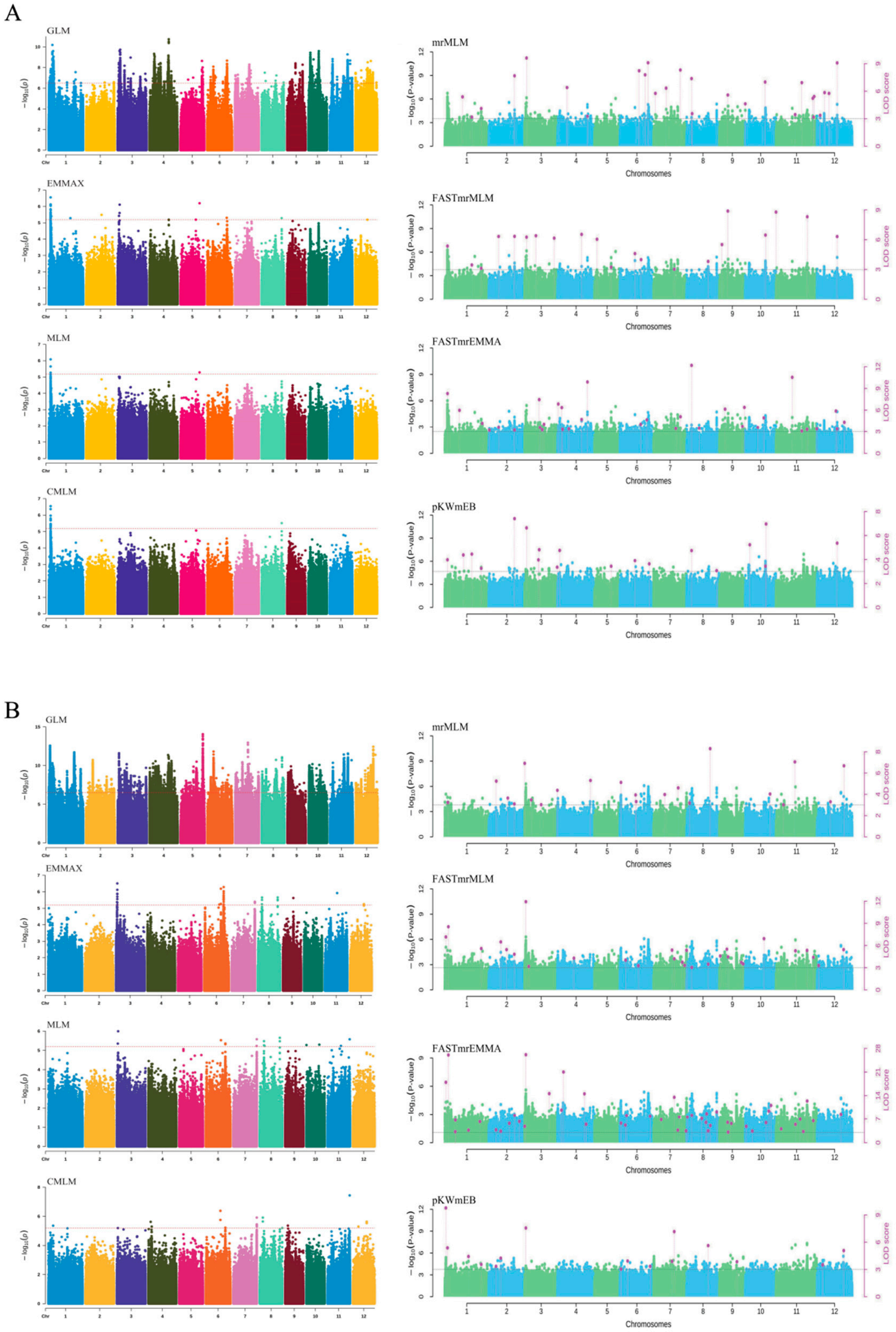
A
GLM
EMMAX
MLM
CMLM
mrMLM
FASTmrMLM
FASTmrEMMA
pKWmEB
Chromosomes
LOD score
B
GLM
EMMAX
MLM
CMLM
mrMLM
FASTmrMLM
FASTmrEMMA
pKWmEB
Chromosomes
LOD score

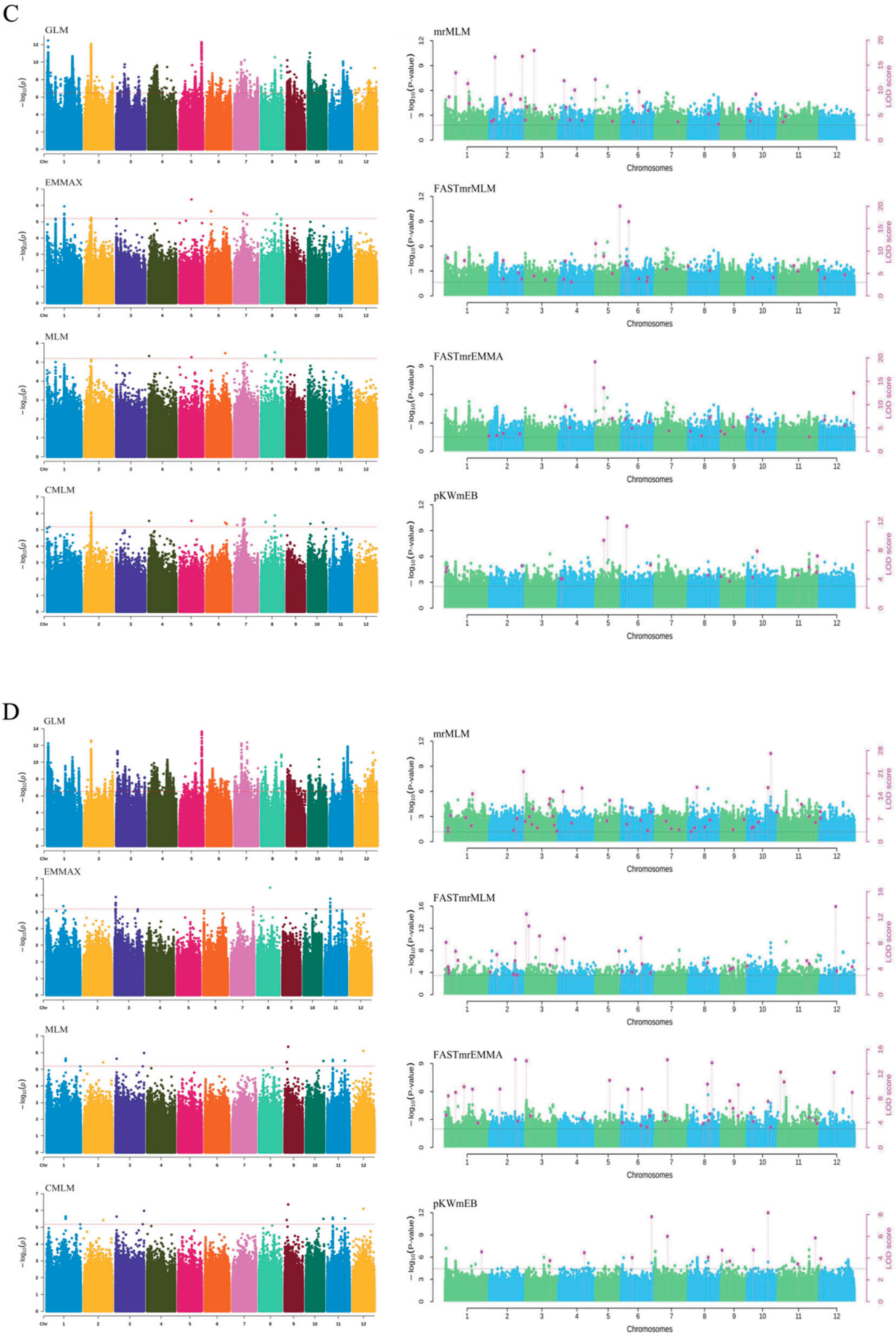
C
GLM
EMMAX
MLM
CMLM
mrMLM
FASTmrMLM
FASTmrEMMA
pKWmEB
Chromosomes
LOD score
D
GLM
EMMAX
MLM
CMLM
mrMLM
FASTmrMLM
FASTmrEMMA
pKWmEB
Chromosomes
LOD score

E

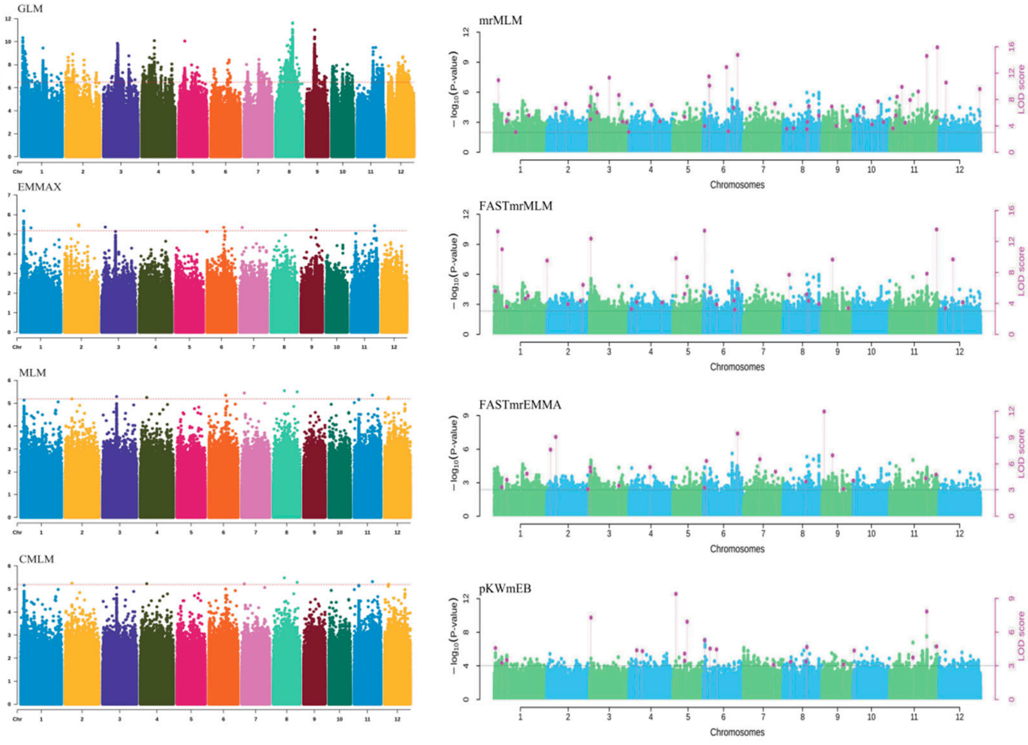
GLM
EMMAX
MLM
CMLM
mrMLM
FASTmrMLM
FASTmrEMMA
pKWmEB
Chromosomes
LOD score
Chr

F

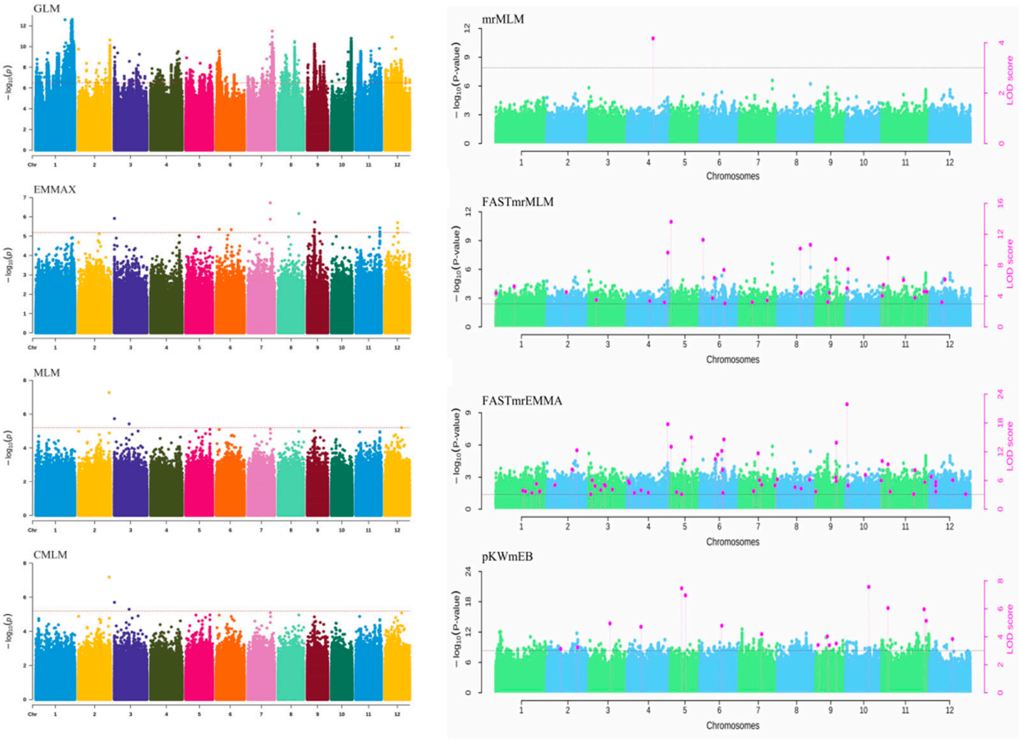
GLM
EMMAX
MLM
CMLM
mrMLM
FASTmrMLM
FASTmrEMMA
pKWmEB
Chromosomes
LOD score
Chr

G

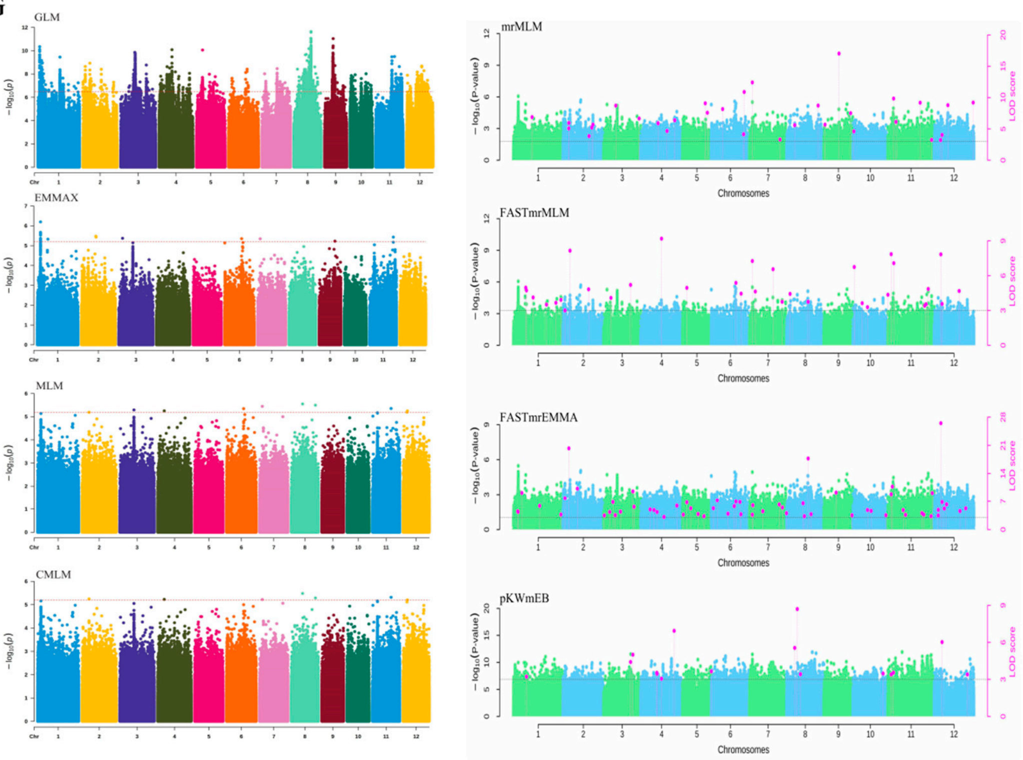

H

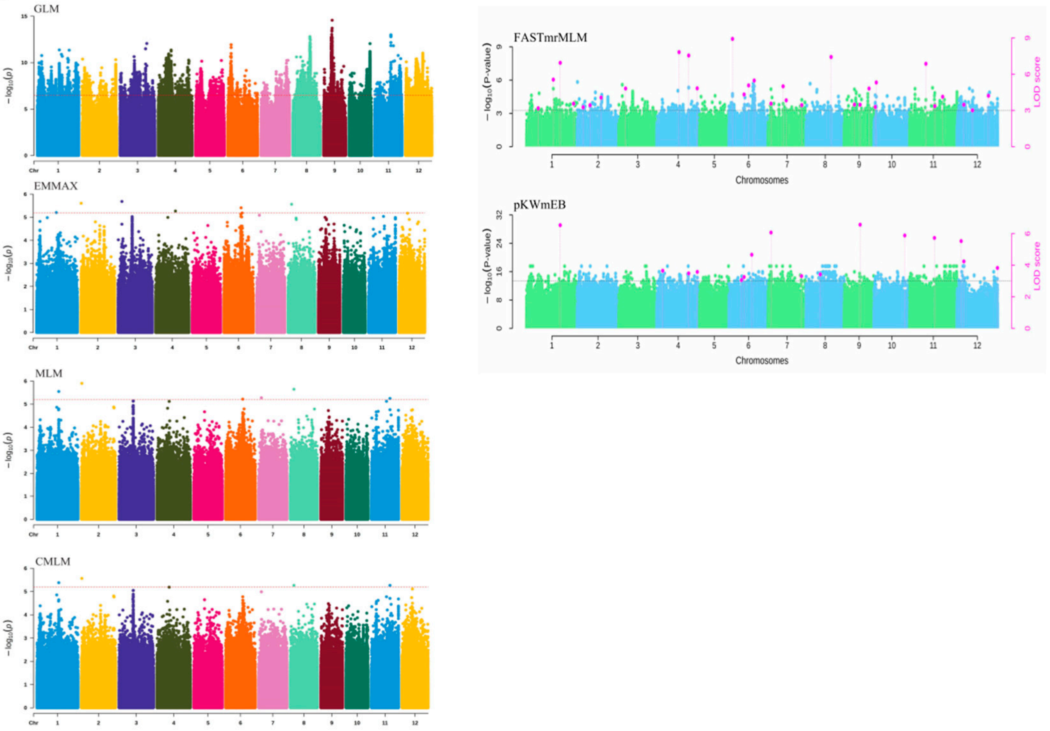

Supplementary Figure S1: Manhattan plots for grain/leaf lysine content associated QTNs using GLM, MLM-based single-locus (MLM-SL), and mrMLM-series multi-locus (mrMLM-ML) models. Manhattan plots for grain/leaf lysine content in Grain_env1_r1 (A), Grain_env1_r2 (B), Grain_env2_r1(C), Grain_env2_r2 (D), Grain_BLUP (E), Leaf_env3_r1 (F), Leaf_env3_r2 (G), and Leaf_BLUP (H)

associated QTNs. MLM-SL includes EMMAX, MLM, and CMLM model. mrMLM-ML includes mrMLM, FASTmrMLM, FASTmrEMMA, and pKWmEB. Black horizontal lines in the Manhattan plots represent the genome-wide significant threshold.

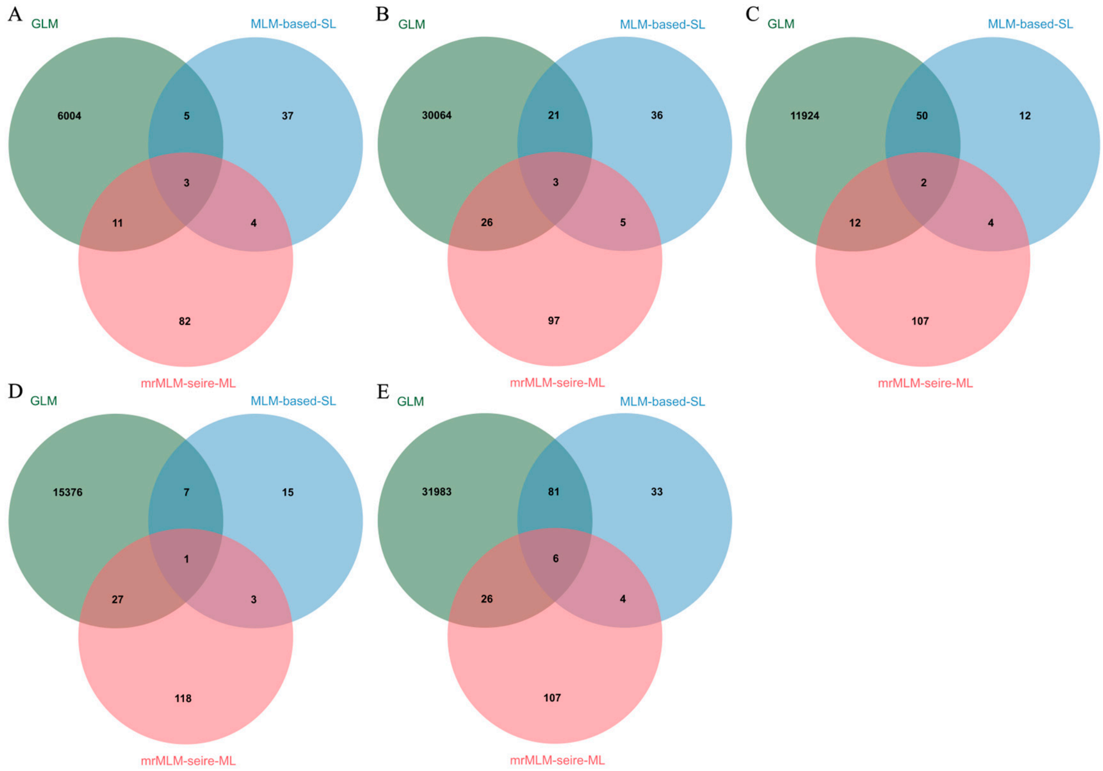

Supplementary Figure S2: Venn diagram of grain lysine associated QTNs using three GWAS models (GLM, MLM-SL, and mrMLM-ML). QTNs detected using five grain lysine content datasets Grain_env1_r1 (A), Grain_env1_r2 (B), Grain_env2_r1 (C), Grain_env2_r2 (D), and Grain_BLUP (E).

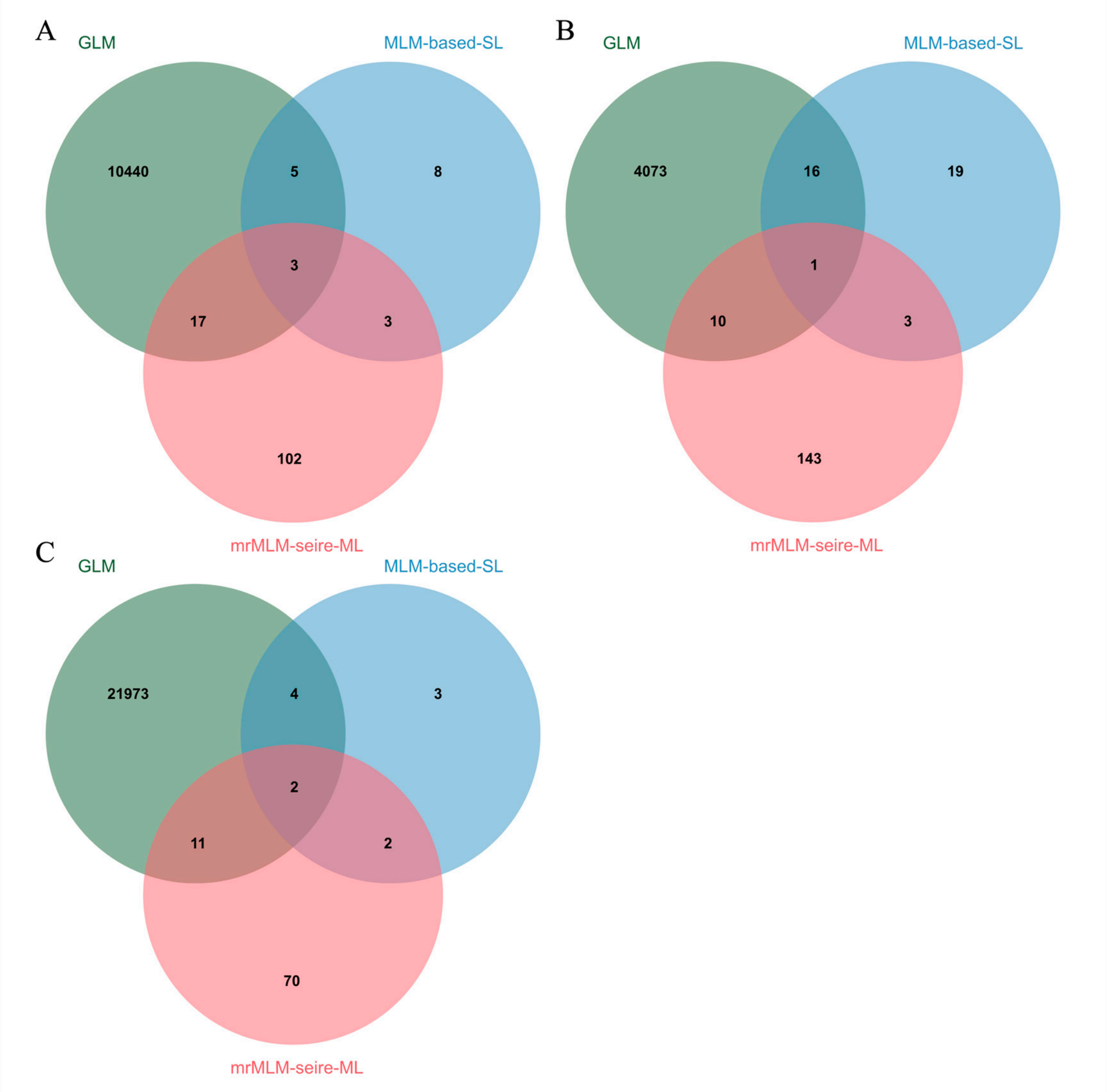

Supplementary Figure S3: Venn diagram of leaf lysine associated QTNs using three GWAS models (GLM, MLM-SL, and mrMLM-ML). QTNs detected using three leaf lysine content datasets Leaf_env3_r1 (A), Leaf_env3_r2 (B), and Leaf_BLUP (C).

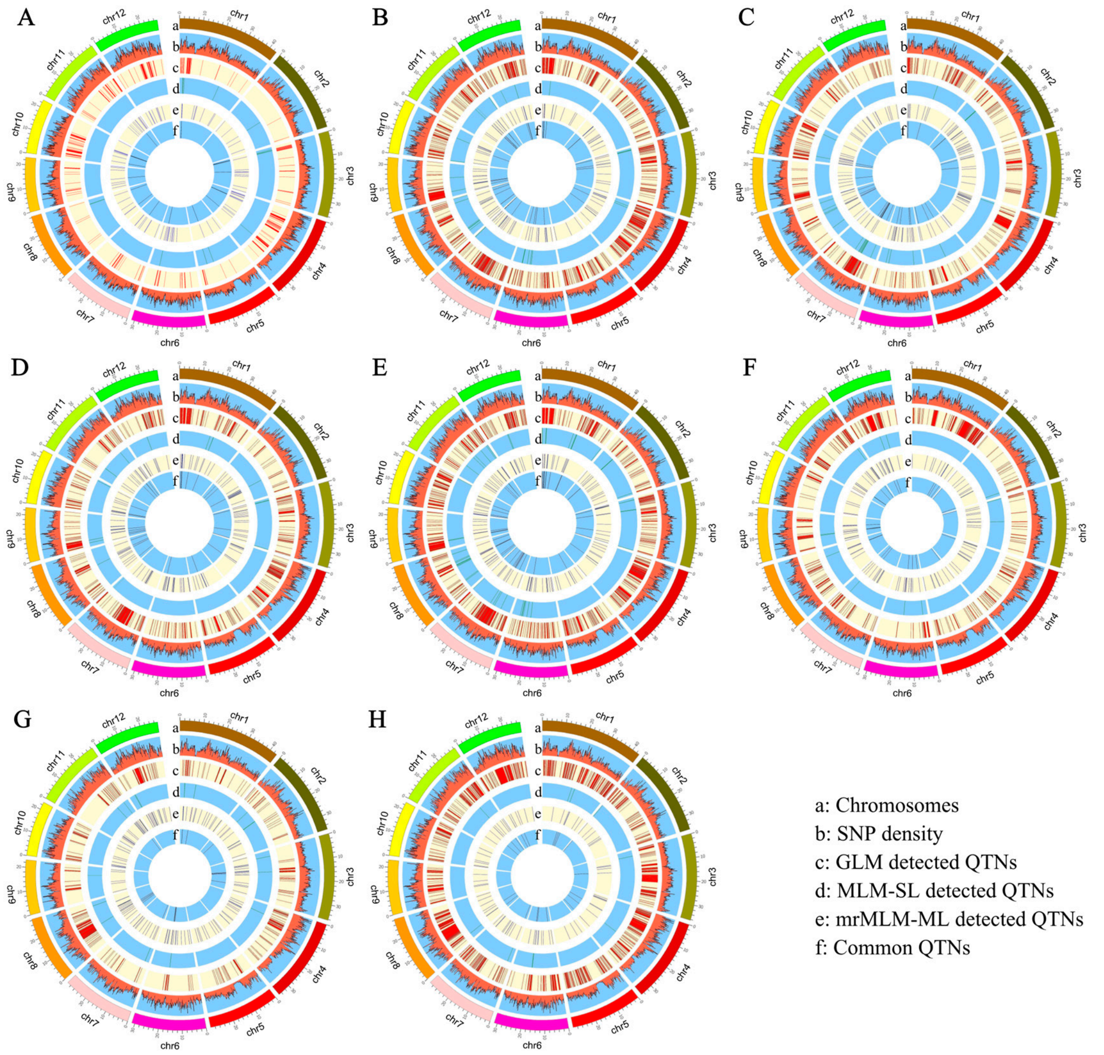

Supplementary Figure S4: Circos map of QTNs and common QTNs in rice genome identified from the grain/leaf lysine content datasets. Grain_env1_r1 (A), Grain_env1_r2 (B), Grain_env2_r1 (C), Grain_env2_r2 (D), and Grain_BLUP (E) for grain lysine content, Leaf_env3_r1 (F), Leaf_env3_r2 (G), and Leaf_BLUP (H) for leaf lysine content. Track a: 12 rice chromosomes; Track b: heatmap of SNP density with bin sizes of 0.1 Mb; Track c: GLM detected QTNs; Track d: MLM-based single-locus model (MLM-SL) detected QTNs; Track e: mrMLM-series multi-locus model (mrMLM-ML) detected QTNs; Track f: common QTNs detected by two or more GWAS models.

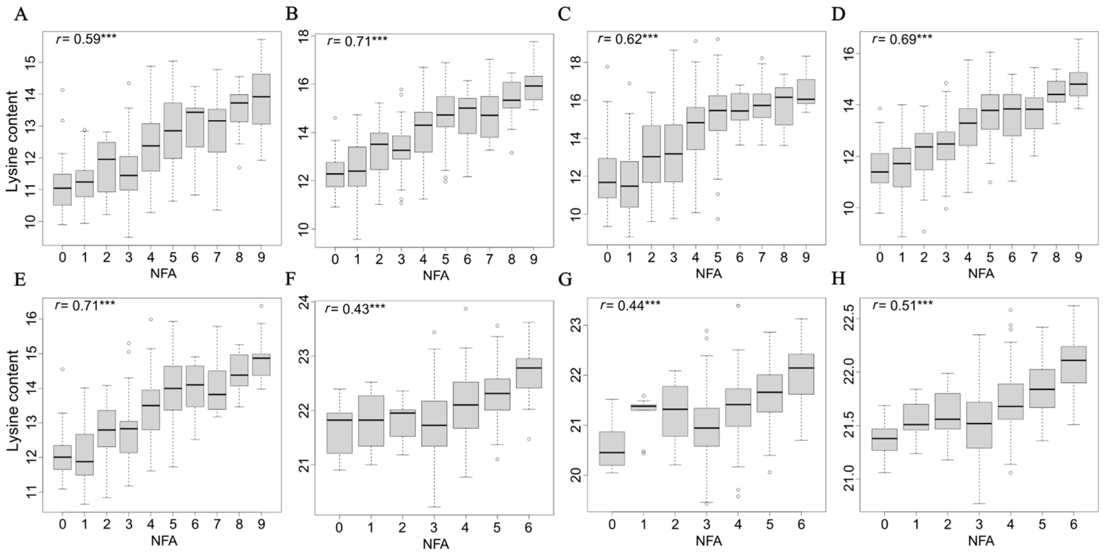

Supplementary Figure S5: Box plots illustrating the relationship between the number of favorable alleles (NFA) and lysine accumulation for five grain and three leaf lysine content datasets. The grain lysine Grain_env1_r1, Grain_env1_r2, Grain_env2_r1, Grain_env2_r2, and the best linear unbiased prediction (BLUP) datasets (A-E), the leaf lysine Leaf_env3_r1, Leaf_env3_r2, and BLUP datasets (F-H). ***indicates statistical significance at the 0.1% probability level.

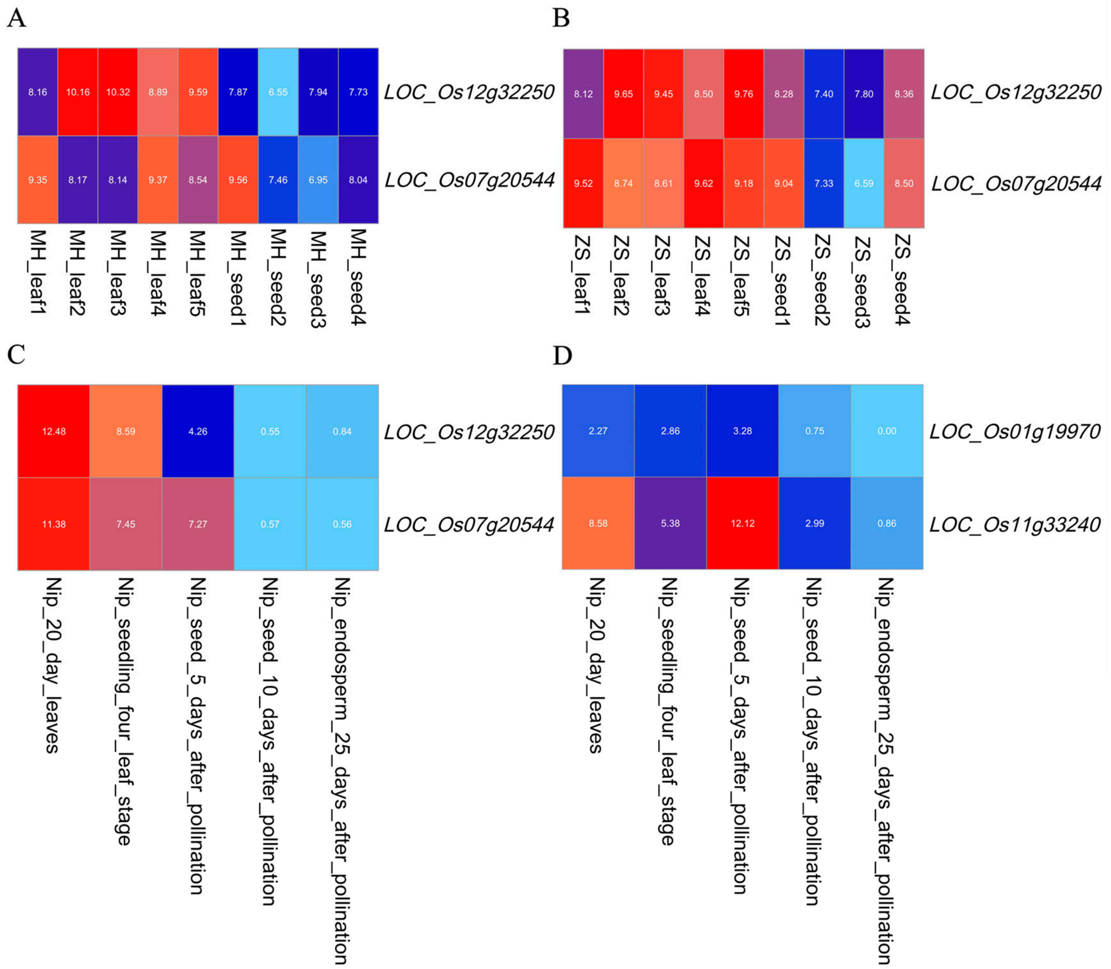

Supplementary Figure S6: Heat map of expression levels of the key genes *LOC_Os12g32250, LOC_Os07g20544, LOC_Os01g19970, and LOC_Os11g33240.* Heat map of expression level of the key genes *LOC_Os12g32250* and *LOC_Os07g20544* in grain and leaf tissue of Nipponbare (A), Minghui 63 (B), and Zhenshan 97 (C) varieties. Heat map of expression level of the key genes *LOC_Os01g19970* and *LOC_Os11g33240* in grain and leaf tissue of Nipponbare (D). The red indicates a high expression, and the blue represents a low expression.

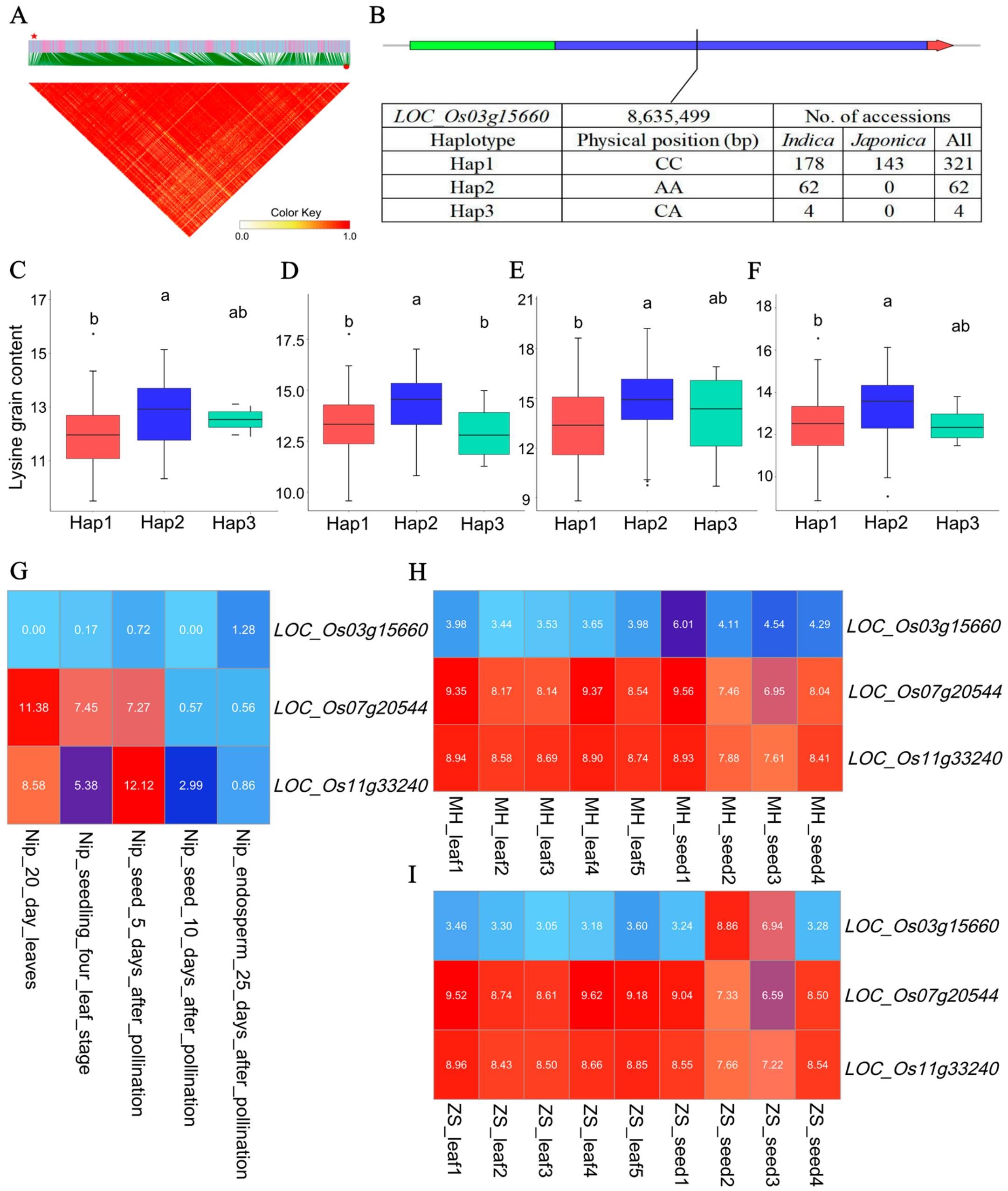

Supplementary Figure S7: Analyses of the key candidate gene *LOC_Os03g15660* related to lysine content in rice. (A) Local linkage disequilibrium block analysis, red star and red dot indicate *LOC_Os03g15660* and QTN-sf0308698430 locus. (B) Three haplotypes of *LOC_Os03g15660* and their distribution in *indica* and *japonica* accessions. The blue, red, and green boxes represent the coding sequence (CDS), five prime UTR, and three prime UTR of a gene, respectively. Haplotypic variation and lysine content analysis of *LOC_Os03g15660* in 387 rice accessions in Grain_env1_r1 (C), Grain_env1_r2 (D), Grain_env2_r1 (E), and Grain_env2_r2 (F) content datasets. Different letters indicate statistically significant differences at the 5% probability level in LSD test. Heat map of expression level of the key genes *LOC_Os03g15660, LOC_Os07g20544, and LOC_Os11g33240* in grain and leaf tissue of Nipponbare (G), Minghui 63 (H) and Zhenshan 97 (I) varieties. The red indicates a high expression, and the blue represents a low expression.
